# The progressive ankylosis gene product ANK regulates extracellular ATP levels in primary articular chondrocytes

**DOI:** 10.1186/ar4337

**Published:** 2013-10-17

**Authors:** Ann K Rosenthal, Claudia M Gohr, Elizabeth Mitton-Fitzgerald, Megan K Lutz, George R Dubyak, Lawrence M Ryan

**Affiliations:** 1 Rheumatology Section, cc-111 W, Zablocki VA Medical Center, 5000 W. National Ave, Milwaukee, WI 53295-1000, USA; 2 Rheumatology Division, Department of Medicine, Medical College of Wisconsin, Milwaukee, WI, USA; 3 Department of Physiology and Biophysics, Case Western Reserve University, Cleveland, OH, USA

## Abstract

**Introduction:**

Extracellular ATP (eATP) is released by articular chondrocytes under physiological and pathological conditions. High eATP levels cause pathologic calcification, damage cartilage, and mediate pain. We recently showed that stable over-expression of the progressive ankylosis gene product, ANK, increased chondrocyte eATP levels, but the mechanisms of this effect remained unexplored. The purpose of this work was to further investigate mechanisms of eATP efflux in primary articular chondrocytes and to better define the role of ANK in this process.

**Methods:**

We measured eATP levels using a bioluminescence-based assay in adult porcine articular chondrocyte media with or without a 10 minute exposure to hypotonic stress. siRNAs for known ATP membrane transporters and pharmacologic inhibitors of ATP egress pathways were used to identify participants involved in chondrocyte eATP release.

**Results:**

eATP levels increased after exposure to hypotonic media in a calcium-dependent manner in monolayer and 3-dimensional agarose gel cultures (*p* < 0.001). A potent transient receptor potential vanilloid 4 (TRPV4) agonist mimicked the effects of hypotonic media. ANK siRNA suppressed basal (*p* < 0.01) and hypotonically-stressed (*p* < 0.001) ATP levels. This effect was not mediated by altered extracellular pyrophosphate (ePPi) levels, and was mimicked by the ANK inhibitor, probenecid (*p* < 0.001). The P2X7/4 receptor inhibitor Brilliant Blue G also suppressed eATP efflux induced by hypotonic media (*p* < 0.001), while ivermectin, a P2X4 receptor stimulant, increased eATP levels (*p* < 0.001). Pharmacologic inhibitors of hemichannels, maxianion channels and other volume-sensitive eATP efflux pathways did not suppress eATP levels.

**Conclusions:**

These findings implicate ANK and P2X7/4 receptors in chondrocyte eATP efflux. Understanding the mechanisms of eATP efflux may result in novel therapies for calcium crystal arthritis and osteoarthritis.

## Introduction

ATP is a key energy-storing compound found in millimolar concentrations inside healthy cells [1]. Most cell types release ATP to the extracellular space under both physiologic and pathologic conditions [1]. In articular cartilage, low levels of extracellular ATP (eATP) transduce mechanical signals [2]. Higher levels of eATP produce pathologic calcium crystal formation such as that seen with calcium pyrophosphate (CPP) and basic calcium phosphate (BCP) crystal deposition in cartilage [3]. eATP also induces production of catabolic mediators such as prostaglandins [4], and activates nociceptive receptors inducing pain [5]. Some of these effects are mediated through purinergic receptors. However, as eATP belongs to the danger-associated molecular pattern (DAMP) family of innate immune signals, it may also contribute to cartilage damage through this mechanism [6,7]. While processes that regulate ATP efflux may be logical therapeutic targets in common degenerative cartilage diseases, surprisingly little is known about transport mechanisms of ATP across the chondrocyte cell membrane.

We recently showed that stable over-expression of the progressive ankylosis gene product (ANK) dramatically increases eATP levels in articular chondrocytes [8]. ANK is a 492 amino acid multipass transmembrane protein originally described as the mutated protein in *ank/ank* mice [9]. Considerable evidence supports its role in extracellular pyrophosphate (ePPi) transport [9,10]. ePPi is a key regulator of pathologic mineralization in cartilage and other tissues. ePPi can be generated from eATP through the action of ecto-enzymes with nucleoside triphosphate pyrophosphohydrolase (NTPPPH) activity, such as ENPP1. Because there is ample ENPP1 activity in normal cartilage to convert all available NTP to NMP and PPi, substrate availability is the rate-limiting step in this reaction [11]. We recently demonstrated that chondrocyte eATP and ePPi elaboration were coordinately regulated [8], supporting a major role for eATP in ePPi production by cartilage. Thus, delineating mechanisms of eATP efflux in cartilage may lead to the identification of novel modulators of ePPi production.

Whether ANK itself may act as an ATP transporter in chondrocytes is not known. Our initial studies involved stable over-expression of ANK, but did not investigate whether over-expression could indirectly increase ATP efflux, for example, by altering the chondrocyte phenotype or affecting levels of eATP metabolizing ecto-enzymes. Structural studies of ANK protein make it unlikely that ANK itself, at least in its monomeric form, is capable of providing a channel of adequate size to accommodate ATP (unpublished observation, C. J. Williams). Thus, the possibility that ANK regulates a known mechanism of cellular ATP export warrants investigation.

Four classic ATP membrane transport mechanisms have been described to date [1]. Hemichannels, composed of either connexin or pannexin proteins, mediate ATP release in many cell types and have been implicated in chondrocyte ATP efflux [12]. Vesicular transport of ATP is best characterized in nerve cells, where ATP is packaged along with other neurotransmitters for rapid release upon cell activation [13]. Vesicular transport of ATP has also been observed in osteoblasts [14]. Two types of molecularly undefined ATP transport channels also exist. Maxianion channels are typically identified by patch clamp experiments, and can be inhibited by anion transport inhibitors and gadolinium [15]. Volume-sensitive outwardly rectifying anion channels (VSOR) or volume-sensitive organic osmolyte and anion channels (VSOAC) are widely expressed channels that rapidly develop after cell swelling. While pharmacologic inhibitors are often used to differentiate between various ATP release mechanisms, interpretations of inhibitor experiments are complicated by considerable overlap in the actions of these agents and anomalous inhibitor responses when multiple transport mechanisms are present in one cell type [1,16].

The ionotropic P2X purinergic receptors, P2X7 and P2X4, have also been implicated in eATP release [17]. These complex receptors respond to stimuli by rapidly opening cation channels and initiating cell signaling. In many cell types, P2X7 and P2X4 receptor channels also comprise or regulate pores capable of transporting molecules as large as 900 Da [18]. P2X7 may co-localize with pannexin proteins, and in some cases hemichannel inhibitors block the activity of the P2X7-regulated large pore [16,19]. P2X7 homotrimeric channels can directly interact with P2X4 homotrimeric channels with consequent changes in trafficking and function of these receptors [20]. Whether purine receptors participate in chondrocyte ATP efflux is not fully understood.

ATP release in cartilage is modulated by mechanical stimuli such as tissue compression and by changes in osmotic pressure. These stimuli are linked by similar effects on membrane tension, and often share signaling pathways [21]. Membrane proteins such as the transient receptor potential vanilloid 4 (TRPV4) may participate in the response to these stimuli [22]. Several studies demonstrate increased ATP efflux in chondrocytes subjected to mechanical compression [12,23]. Exposure to osmotic stress is a commonly used model to study ATP efflux [24,25]. Osmotic changes are particularly relevant in cartilage, where mechanical forces repetitively force water in and out of the highly charged extracellular matrix. Normal chondrocytes reside in a hyperosmolar environment (350 to 480 mOsm/L), which is reduced in well-established osteoarthritis (OA) to 280 to 350 mOsm/L [26,27]. The effects of an osmotic challenge on eATP release in articular chondrocytes and the signals involved in this process remain poorly characterized.

The purpose of this work was to further identify mechanisms of basal and hypotonically stressed eATP efflux in primary articular chondrocytes and characterize the involvement of ANK in these processes.

## Methods

### Materials

Unless otherwise specified, all reagents were from Sigma Aldrich Chemical Co. (St Louis, MO, USA): ^10^panx1 and its scramble control, the P2X7 inhibitors A438079 and AZ10606120, and GSK1016790A, were obtained from Tocris (Ellisville, MO, USA).

### Chondrocyte cultures

Primary hyaline articular chondrocytes were isolated from knee joints of 3- to 5-year-old pigs as previously described [28]. Knee cartilage was obtained from pigs slaughtered at a local abattoir and was used in accordance with guidelines from the Subcommittee on Animal Use of the Research and Development Committee of the Zablocki VA Medical Center. Chondrocytes were plated in high-density short-term monolayer cultures and used within 3 days of plating. DMEM (low glucose, 335 ± 30 mOsm/L) was used for all experiments. Initial experiments were repeated with chondrocytes embedded in 2% agarose constructs. To embed chondrocytes, freshly digested cells (5 × 10^6^ cells/ml) were mixed 1:1 with 4% agarose in Hank’s Balanced Salt Solution (HBSS). One hundred μl of warm agarose containing cells were added to each well of a 96-well plate and allowed to solidify. After solidification, 150 μl of DMEM were added to each well.

### eATP measurements

Media were removed from chondrocytes plated in 96-well clear-bottom black plates, and replaced with fresh serum-free DMEM with or without ATP modulators or other additives. After 30 minutes aliquots of media were removed and replaced with an equal volume of sterile water to expose cells to hypotonic media or fresh DMEM as a control. After 10 minutes eATP levels were measured in the media using the Sigma ATP Assay Mix (FLAAM) and read in a BioTek® Synergy HT plate reader (BioTek®, Winooski, VT, USA). The osmolarities of all media preparations including those with and without inhibitors and other additives were measured with an Osmette osmometer (Precision Systems, Natick, MA, USA). Media osmolarities were as follows: undiluted media 362 to 302 mOsm/L, 15% H_2_O 282 to 249 mOsm/L, 35% H_2_O 216 to 192 mOsm/L, and 50% H2O 166 to 143 mOsm/L. No media additives, except for water, altered media osmolarity more than 10% (data not shown). We chose to use 10 to 50% water as an osmotic challenge, as this level of osmotic stress typically induces eATP release in other cell types [29,30]. Each culture additive and osmotic condition was tested for effects on the ATP standard curve. If effects were noted, as they were in the case of sodium pyrophosphate (NaPPi) and Brilliant Blue G (BBG), calculated ATP levels were adjusted accordingly.

### ATP metabolizing ecto-enzyme activities

Specific activities of the ecto-enzymes that metabolize ATP were measured, as changes in these enzyme activities could affect eATP levels without altering transport. NTPPPH activity was measured using 2 mM p-nitrophenol thymidine monophosphate (PNPMP) as a substrate. Briefly, the media were removed and replaced with PNPMP in HBSS. The cells were incubated for 2 h at 37°C and the reaction was stopped with the addition of 0.1 N NaOH. The absorbance was measured at 410 nm using a Biotek plate reader. Activity of the phosphate-generating enzyme, 5′ nucleotidase (5′ NT), was determined with a kit used according to manufacturer’s directions (BQ Kits, San Diego, CA, USA) [31]. Alkaline phosphatase activity was measured using p-nitrophenol phosphate (PNPP) as a chromogenic substrate. Cells were lysed in 0.9% saline with 0.2% Triton™ x-100. Equal volumes of alkaline buffer solution and PNPP were added and incubated for 15 minutes at 37°C. The reaction was stopped with 0.05 N NaOH and absorbance was measured as described above. All results were corrected for protein levels in the samples using the Lowry assay.

### Calcium dependence

To determine if the ATP response to a hypotonic challenge was calcium dependent, we exposed chondrocytes to the calcium ionophore, A23187. Bis-N,N,N',N'-tetraacetic acid-AM (BAPTA-AM) was used to buffer changes in intracellular calcium flux as described [32,33]. We also explored the ability of the TRPV4 agonist GSK1016790A to stimulate eATP efflux.

### Cell toxicity

All culture additives were tested for toxicity using the 3-(4,5-dimethylthiazol-2-yl)-2,5-diphenyltetrazolium bromide (MTT) formazan assay according to manufacturer’s directions.

### Chondrocyte transfection

Chondrocytes freshly isolated from whole cartilage were nucleofected with siRNA for the protein of interest or non-targeting scramble control with an Amaxa Nucleofection device using program H-020. All silencers were purchased from Life Technologies (Grand Island, NY, USA). Stealth silencers for P2X4 and P2X7 were custom designed using porcine-specific sequences, and ANK silencer (133667) was predesigned and prevalidated. Prior to plating transfected cells, viability was assessed with trypan blue. Transfected chondrocytes were incubated in monolayer cultures for 48 to 72 h prior to RNA isolation, and eATP measurements were performed.

### RNA isolation, reverse transcription and real-time PCR

Total RNA was extracted from chondrocytes using the PureLink Mini RNA kit (Life Technologies). cDNA was synthesized from 1 μg of total RNA using QuantiTect Reverse Transcription kit (Qiagen, Valencia, CA, USA), which includes a genomic DNA elimination step. mRNA expression was measured by quantitative real-time PCR using SYBR Green Master I Mix on the LightCycler 480 Real-Time PCR System (Roche, Indianapolis, IN, USA). Two reference genes were selected for normalization after determining they were stably expressed across samples. After verifying similar amplification efficiencies with a 5-point standard curve, the comparative cycle threshold (Ct) method was used to calculate fold change. Cycling conditions were set as follows: one cycle at 95°C for 10 minutes, 40 cycles of 95°C for 15 seconds, 60°C for 30 seconds, and 72°C for 15 seconds. A melting curve analysis was performed to confirm amplification specificity. The final PCR products were electrophoresed on a 1% ethidium bromide-stained agarose gel to verify the presence of a single band. Primer sequences are available upon request.

### Western blotting

Chondrocyte lysates were loaded onto 10% NuPage® (Invitrogen, Grand Island, NY, USA) Bis-Tris gels. After electrophoresis, proteins were blotted onto poly-(vinylidene) difluoride membranes (Life Technologies). Membranes were blocked in a Tris-buffered saline (TBS)-igepal-5% skim milk buffer for 1 h at room temperature. They were then exposed to antibodies directed against connexin-43 (Abcam, Cambridge, MA, USA), pannexin 1 and 3, ANK, P2X4, P2X7 and TRPV4 (Santa Cruz, Santa Cruz, CA, USA) at 1:1,000 to 10,000 dilution for 1.5 to 24.0 h. After washing, the membranes were exposed to peroxidase-labeled goat anti-rabbit IgG (H + L) or rabbit anti-goat for 1 h (1:2,500) (Life Technologies). Both the primary and secondary antibody exposures were performed in a TBS-igepal-0.5% skim milk buffer. SuperSignal® West Femto Maximum Sensitivity Substrate (Pierce, Rockford, IL, USA) was used to visualize immunoreactive protein bands.

### Prostaglandin E_2_ levels

Prostaglandin E_2_ levels in chondrocyte media were measured using Parameter™ Prostaglandin E_2_ kit (R&D Systems, Minneapolis, MN, USA) according to manufacturer’s directions.

### Statistics

All experiments were repeated a minimum of three times. An individual experiment is considered as the data derived from a chondrocyte culture isolated from one set of pig knees. The number of replicates (n) within experiments was typically eight in each group. As ATP levels failed to satisfy criteria for parametric variables, the non-parametric Mann–Whitney *U*-test was used to determine the statistical significance of the inhibitor effects on eATP release. Parametric outcomes were evaluated with the unpaired Student *t*-test. Statistical significance was set at *P* <0.05.

## Results

### eATP levels in chondrocyte media are increased by exposure to hypotonic conditions, and proteins implicated in eATP efflux are present in chondrocytes

**Figure 1:**
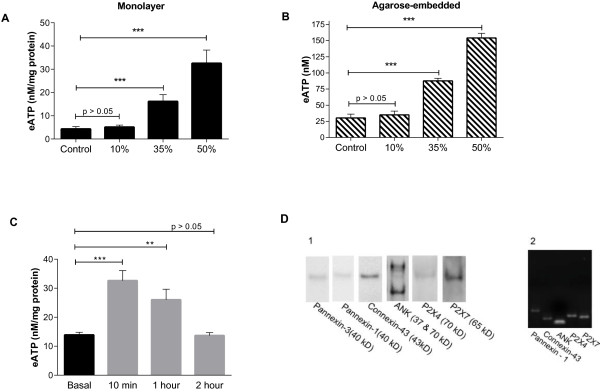
**Effect of a hypotonic challenge on eATP levels in media from monolayer and agarose cultures and characterization of putative ATP transport participants. (A)** Normal adult porcine chondrocytes were plated in high-density short-term monolayer cultures. Media were replaced with fresh media (control) or identical quantities of media containing increasing percentages (volume/volume) of H_2_O. eATP levels were measured 10 minutes post-treatment. Bars represent mean ± standard error **(A-C)**. eATP levels were increased by 35 and 50% H_2_0 compared to an undiluted media control (n = 8;^***^P <0.001). **(B)** Chondrocytes were embedded in 2% agarose. Media were replaced with fresh media (control) or identical quantities of media containing increasing percentages (volume/volume) of H_2_O. eATP levels were measured 10 minutes after the media change. eATP levels were increased by 35 and 50% H_2_0 compared to undiluted media control (n = 8;^***^P <0.001). **(C)** Chondrocytes were exposed to hypotonic media (gray bars) or isotonic media (black bars) for various lengths of time and eATP levels were measured. eATP levels were increased compared to unchallenged control at 10 minutes and 1 h (n = 8;^***^P <0.001, ^**^P <0.01). **D**. Chondrocyte protein extracts were run in western blots using specific antibodies against pannexin 1 and 3, ANK, P2X7 and P2X4. Total RNA was reverse transcribed and analyzed for mRNA for pannexin 1, ANK, P2X7 and P2X4 as described. The final PCR products were electrophoresed on a 1% ethidium bromide-stained agarose gel. Due to absence of a sequence for porcine pannexin-3, RT-PCR for pannexin-3 was not performed.

Baseline eATP levels in chondrocyte-conditioned media were consistently measureable, but absolute values varied considerably between experiments. Exposure to more than 35% water significantly increased eATP levels after 10 minutes in a dose-dependent manner as shown in the representative experiment in Figure 1A (*P* <0.001). We demonstrated an identical dose response to a hypotonic challenge in chondrocytes embedded in an agarose matrix (*P* <0.001) (Figure 1B). Levels fell back to baseline levels 2 h after a hypotonic challenge (Figure 1C). These findings support the physiologic relevance of the monolayer culture system. For all further experiments, monolayer cultures were utilized, and exposure to 35% water for 10 minutes was chosen as the standard hypotonic challenge. To characterize the potential participants in eATP efflux in primary chondrocytes, we ensured that pannexin-1 and -3, connexin 43, ANK, P2X7, and P2X4 were present using western blotting and reverse transcription (RT)-PCR (Figure 1D).

### The response to a hypotonic challenge is calcium-dependent and mimicked by a specific TRPV4 agonist

**Figure 2:**
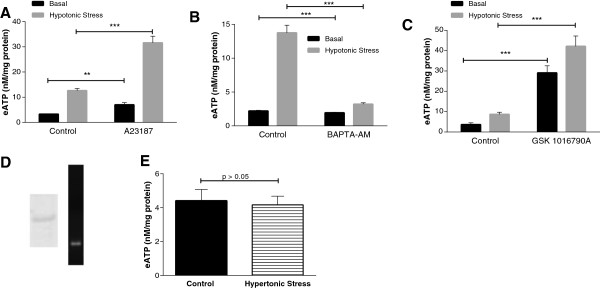
**Roles of Ca**^**2+ **^**signaling and transient receptor potential vanilloid 4 (TRPV4) channels in hypotonically-induced release of eATP from chondrocytes.** For the following experiments, chondrocytes were incubated with each additive for 30 minutes in 100 μl of media with no additives acting as a control. A hypotonic challenge was initiated by removing 35 μl of media, replacing it with 35 μl of H_2_O. eATP levels were measured after 10 minutes. Bars represent mean ± standard error. Under control conditions, a hypotonic challenge consistently increases (eATP). **(A)** Chondrocytes were incubated with 100 μM A23187, which increased eATP levels (n = 8; ^**^P <0.01, ^***^P <0.001). **(B)** Chondrocytes were incubated with 10 μM bis-N,N,N',N'-tetraacetic acid-AM (BAPTA-AM), which decreased eATP levels after hypotonic challenge compared to control without BAPTA-AM (n = 8; ^***^P <0.001). **(C)** Chondrocytes were incubated with 100 nM GSK1016790A, which increased eATP levels in the basal state and after a hypotonic challenge compared to a control without GSK 1016790 (n = 8; ^***^P <0.001). **(D)** Chondrocyte protein extracts were run in western blots with TRPV4 antibody (leftpanel). mRNA for TRPV4 was detected with RT-PCR using a specific primer sequence and electrophoresed on a 1% ethidium bromide-stained agarose gel ( right panel). **(E)** Chondrocytes were incubated with control media (black bars) or media containing an additional 100 mM NaCl (gray bars) for 10 minutes after which eATP levels were measured. A hypertonic challenge did not increase eATP levels compared to the basal state (n = 8; *P* >0.05).

As shown in Figure 2A, the calcium ionophore A23187 stimulated eATP efflux and mimicked the effects of exposure to hypotonic media (*P* <0.001). As calcium ionophores have additional cellular effects, we also investigated the actions of BAPTA-AM, which buffers intracellular calcium. BAPTA-AM reduced the effect of the hypotonic challenge on eATP efflux (*P* <0.001), supporting a role for calcium (Figure 2B). BAPTA-AM had no effect on basal levels of eATP. TRPV4 is an osmotically-sensitive non-selective cation channel that has been implicated in ATP efflux in other cell types [34] and is present in chondrocytes (Figure 2D). Figure 2C shows that the TRPV4 agonist, GSK1016790A, mimics the effects of a hypotonic challenge (*P* <0.001). A role for TRPV4 in mediating the effects of hypotonicity is further supported by the lack of response to a hypertonic challenge (Figure 2E), a property characteristic of TRPV4-mediated effects [22].

### ANK siRNA suppressed basal and hypotonically stressed eATP levels in chondrocyte cultures

**Figure 3:**
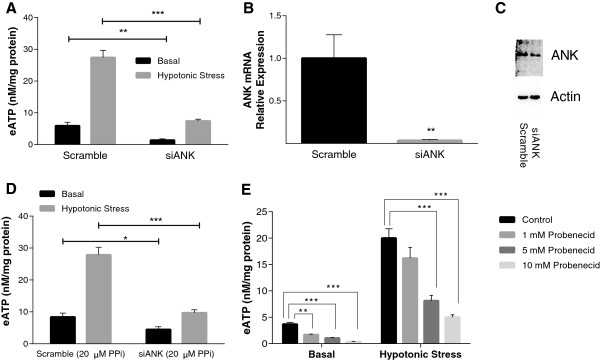
**Role of progressive ankylosis gene product (ANK) in basal and hypotonically stressed eATP release from chondrocytes.** For the following experiments, a hypotonic challenge was initiated by removing 35 μl of media, replacing it with 35 μl of H_2_O. eATP levels were measured after 10 minutes. Under control conditions, hypotonic challenge consistently increases eATP. Bars represent mean ± standard error; n = 8 for all analyses. **(A)** Chondrocytes were transfected with siRNA for ANK or scramble control. After 48 h, cells were exposed for 10 minutes to hypotonic (gray bars) or isotonic (black bars) media and eATP levels were measured. siANK suppressed eATP levels under basal and hypotonically challenged conditions (^**^P <0.01). **(B**, **C)** In parallel cultures at 48 h, mRNA and protein were isolated to assess levels of ANK mRNA (^**^P <0.01) and protein. Western blots compared effects on ANK versus actin. **(D)** Transfected chondrocytes (as described above) were exposed to media containing no additives (control) or 20 μM PPi in 100 μl of media for 30 minutes with or without hypotonic challenge. The addition of PPi did not alter eATP levels compared to controls without PPi (*P* >0.05). **(E)** Chondrocytes were treated with no additives (control), 1 mM probenecid, 5 mM probenecid or 10 mM probenecid (as described above). After the hypotonic challenge eATP levels with 1 mM probenecid were not statistically different from the control (*P* >0.05): 5 to 10 mM probenecid decreased eATP levels in basal and hypotonically challenged states (^**^P <0.01, ^***^P <0.001).

We have previously shown that over-expression of the putative ePPi transporter ANK in chondrocytes resulted in a 10-fold increase in eATP levels compared to controls transfected with an empty viral vector [8]. To extend these findings, we explored the effect of specifically reducing ANK levels on eATP levels in chondrocyte media. eATP levels were suppressed in chondrocytes treated with ANK siRNA compared to those treated with a scramble control (*P* <0.001) (Figure 3A), without alterations of ecto-enzyme activities or cell viability (data not shown). ANK mRNA (*P* <0.01) (Figure 3B) and protein levels (Figure 3C) were significantly reduced in ANK siRNA-treated chondrocytes. To ensure that reductions in eATP in ANK-silenced cells were not indirectly due to decreases in ePPi levels, we added back 10 to 100 μM NaPPi to the media of ANK-silenced cells and measured eATP levels. NaPPi did not alter the pH of the media, which remained at pH 7.4. As shown in the representative experiment in Figure 3D, the presence of exogenous PPi did not restore eATP levels in ANK-silenced cells towards levels seen in the scramble control. However, there were small increases in eATP levels in the presence of added ePPi seen across groups, which were not statistically significant. These data suggest that the reduction in eATP seen with ANK silencing is not mediated by changes in ePPi concentrations. Probenecid, which has been shown to inhibit ANK-mediated PPi transport [35], reduced eATP levels in a dose-dependent manner (*P* <0.001) (Figure 3E).

### ANK may act to directly transport ATP or regulate other ATP transport mechanisms

We also designed experiments to test for the presence of classic ATP egress pathways by investigating the effects of inhibitors of these pathways. None of the pharmacologic inhibitors reduced basal eATP levels with the exception of probenecid (data not shown). Table 1 summarizes the effects of these pharmacologic inhibitors on eATP levels measured after a hypotonic challenge. Results are expressed as the fold change in eATP levels after a hypotonic challenge in the presence of the inhibitor compared to the absence of the inhibitor. Despite the expression of hemichannels, including pannexin-1 and connexin-43, by chondrocytes at the protein and mRNA levels (Figure 1D), multiple pharmacological inhibitors known to target hemichannels failed to suppress osmotically induced chondrocyte ATP. The effect of ^10^panx1, a small peptide inhibitor of pannexin-1 hemichannels [36], was indistinguishable from its control peptide at concentrations from 100 to 400 μM. Flufenamic acid (30 to 500 μM) and carbenoxolone (1 to 100 μM) also failed to significantly suppress hypotonically-induced eATP production. Small decreases in eATP levels were seen with vesicular transport inhibitors, including monensin (100 μM) and brefeldin (100 μM), but these failed to achieve statistical significance. Inhibitors implicated in the molecularly undefined maxianion and VSOARC channels such as gadolinium (50 μM) did not effectively decrease eATP levels in the media from osmotically stressed chondrocytes.

**Table 1:** Effects of pharmacologic ATP transport inhibitors on eATP levels, ATP-metabolizing ecto-enzyme activities, and cell toxicity in hypotonically stressed chondrocyte cultures

**Inhibitor**	**Target**	**Dosage**	**Fold change in ATP**	***P***	**N**	**Alk phos**	**NTPPPH**	**5’NT**	**Toxicity (fold change)**
Probenecid	ANK, Hemichannels	5 mM	0.59*	0.004	83	103 ± 2.9	108 ± 6.9	122 ± 24.8	1.31
Monensin	Vesicular	100 uM	0.74	0.097	50	103 ± 1.8	109 ± 5.3	120 ± 20.2	1.49
GdCl_3_	Maxianion	50 uM	0.88	0.309	51	106 ± 6.9	104 ± 2.1	126 ± 11.9	2.21
N-ethylmalemide (NEM)	Vesicular	100 uM	0.89	0.947	57	101 ± 3.2	110 ± 3.6	116 ± 19.3	1.68
Brefeldin	Vesicular	100 uM	0.88	0.739	60	101 ± 3.2	110 ± 3.6	116 ± 19.3	1.68
Carbeneoxolone (CBX)	Hemichannels	5 uM	1.45	0.627	40	103 ± 2.9	109 ± 8.4	109 ± 16.9	3.25
Flufenamic acid (FFA)	Hemichannels	30 uM	1.14	0.545	40	98 ± 6.5	118 ± 6.0	108 ± 14.4	1.33
^10^Panx1	Pannexin-1	100 uM	1.3	0.850	43	105 ± 4.7	137 ± 4.6	115 ± 16.8	1.33
^10^Panx1 Scramble	Pannexin-1	100 uM	1.5	0.256	41	104 ± 6.6	142 ± 8.8	83 ± 10.8	1.27
Brilliant Blue G	P2X7, P2X4	50 uM	0.367*	0.001	24	147 ± 5.9	142 ± 7.4	155 ± 32	1.26
A438079	P2X7	300 nm	2	0.042	24	131 ± 4.4	136 ± 5.7	92 ± 21.9	1.25
AZ10606120	P2X7	10 nM	1.2	0.806	24	123 ± 6.9	124 ± 4.6	104 ± 23.6	1.12

Chondrocytes were exposed to control media with and without various pharmacologic ATP pathway inhibitors for 30 minutes. Aliquots of media were removed and replaced with 35% H_2_0 as described. After 10 minutes, eATP levels were measured in the media. eATP results are expressed as a mean fold change in osmotically challenged eATP levels over control (no inhibitor) conditions; N = number of replicates pooled from three to five experiments: *statistically significantly inhibition of ATP levels compared to controls. In parallel cultures, specific activities of ATP metabolizing enzymes - alkaline phosphatase (Alk phos), nucleoside triphosphate pyrophosphohydrolase (NTPPPH), and 5′ nucleotidase (5' NT) - were measured in the cell layer using standard colorimetric assays. Results are expressed as percent of specific activity of the control (no inhibitor) conditions (means ± standard error, n=6). Cell toxicity was measured with the 3-(4,5-dimethylthiazol-2-yl)-2,5-diphenyltetrazolium bromide (MTT) assay. Results are expressed as a fold increase over control (no inhibitor) conditions. There were no statistically significant changes in levels of Alk phos, NTPPPH, 5’NT activity or cell toxicity in the presence of inhibitors. ANK, progressive ankylosis gene product.

### Possible roles for P2X7 and P2X4 receptor channels in chondrocyte eATP release

**Figure 4:**
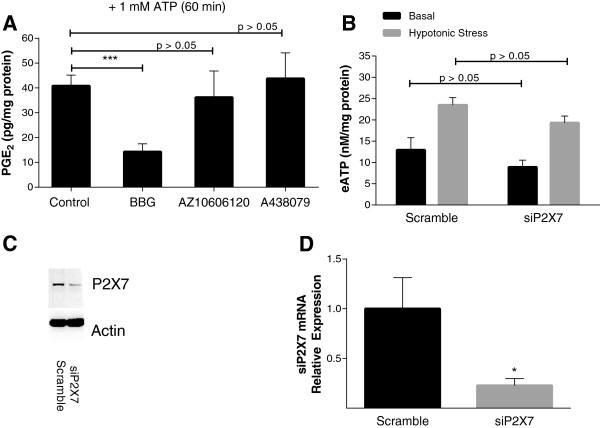
**Role of P2X7 receptors in signaling and eATP efflux by chondrocytes. (A)** Chondrocytes were treated with no additives (control) or P2X7 inhibitors (Brilliant Blue G (BBG), AZ10606120 or A438079) for 1 h in the presence of 1 mM ATP. Prostaglandin E_2_ (PGE_2_) levels in the media were measured using the Parameter™ Prostaglandin E_2_ kit (R&D Systems). Bars represent mean ± standard error. BBG reduced ATP-induced PGE_2_ levels (n = 8; ^***^P <0.001). **(B)** Chondrocytes were transfected with siRNA for P2X7 or a scrambled control. After 48 h, cells were exposed to hypotonic media for 10 minutes (gray bars) or isotonic media (black bars) and eATP levels were measured. Bars represent mean ± standard error. Under control conditions, a hypotonic challenge consistently increases (eATP). No differences in eATP levels were noted in siP2X7-treated chondrocyte media (n = 8; *P* >0.05). In parallel cultures at 48 h, protein and mRNA were isolated from scramble or siRNA-treated cells as described and used to assess levels of P2X7 receptor protein **(C)** and mRNA **(D)**. mRNA levels of P2X7 were suppressed in siRNA-treated cells (^*^P <0.05). Western blots compare the effects of siRNA effects on P2X7 receptor levels versus actin.

**Figure 5:**
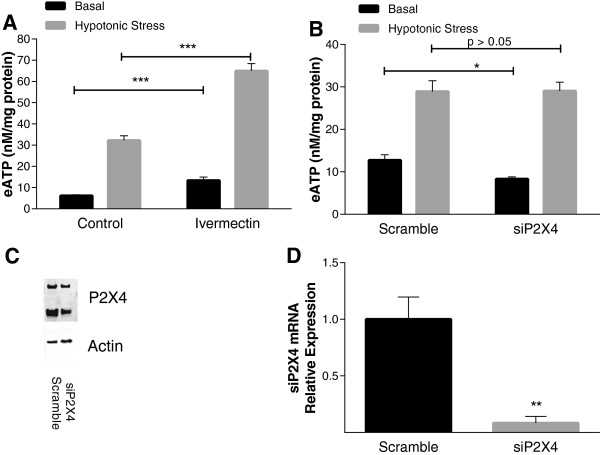
**Role of P2X4 receptors in signaling and eATP release by chondrocytes. (A)** Chondrocytes were treated with no additives (control) or 10 μM ivermectin in 100 μl of media for 30 minutes: 35 μl of media were removed and replaced with 35 μl of media (black bars) or 35 μl of H_2_O (gray bars). eATP levels were measured after 10 minutes. Bars represent mean ± standard error. Under control conditions, a hypotonic challenge consistently increases (eATP). Ivermectin increased eATP levels after a hypotonic challenge (n = 8: ^***^P <0.001). **(B)** Chondrocytes were transfected with siRNA for P2X4 or a scrambled control. After 48 h, cells were exposed to hypotonic media for 10 minutes (gray bars) or isotonic media (black bars) and eATP levels were measured. Bars represent mean ± standard error. eATP levels in P2X4 -silenced chondrocyte media were similar to those in the scramble control media (n = 8; *P* >0.05). In parallel cultures at 48 h, protein and mRNA were isolated from scramble or siRNA-treated cells as described and used to assess levels of P2X4 receptor protein **(C)** and mRNA **(D)**. siRNA for P2X4 decreased P2X4 mRNA (^**^P <0.01). Western blots compare effects on P2X4 receptor versus actin.

The insensitivity of chondrocyte eATP accumulation to multiple inhibitors that target defined ATP release mechanisms was surprising. Although many studies with these inhibitors have been performed in cells that over-express proteins involved in a single ATP transport mechanism pathway, ATP transport mechanisms have been successfully teased out in primary cells using these methodologies [14]. P2X7 receptors may play a direct role in eATP release in some cell types, as the large pore that opens upon P2X7 activation may itself release ATP [17]. P2X4 may also function in this manner [37]. P2X7 and P2X4 receptor protein and mRNA are expressed in primary chondrocytes (Figure 1D). Complexes containing both P2X7 homotrimeric channels and P2X4 homotrimeric channels have been characterized in leukocytes [20,38]. As shown in Table 1, we explored the effects of three different P2X7 receptor inhibitors on eATP release. BBG, which inhibits both P2X4 and P2X7 receptors, significantly suppressed eATP levels after a hypotonic challenge, whereas two specific P2X7 receptor inhibitors, A438079 and AZ10606120, failed to do so. No effects on basal eATP levels were seen with any of these inhibitors (data not shown). To determine whether this pattern correlated with other putative P2X7 receptor-mediated actions, we measured ATP-induced prostaglandin E_2_ (PGE_2_) release from chondrocytes, which is a P2X receptor-dependent effect, and may also be associated with pore formation [39]. Only BBG inhibited PGE_2_ release by chondrocytes (*P* <0.001) (Figure 4A). Moreover, treatment of chondrocytes with siRNA that targeted P2X7 receptors failed to significantly decrease hypotonically-stressed ATP release (Figure 4B) despite causing decreased levels of P2X7 receptor protein (Figure 4C) and mRNA (*P* <0.05) (Figure 4D). The ability of BBG but not A438079, AZ10606120, or P2X7 siRNA to attenuate ATP release suggested involvement of the P2X4 subtype. Among the P2X receptors, P2X4 receptors characteristically respond to ivermectin with increased channel gating and activity. As shown in Figure 5A, ivermectin increased eATP levels in chondrocytes after a hypotonic challenge (*P* <0.001). Although we were able to effectively decrease levels of P2X4 protein (Figure 5C) and mRNA (*P* <0.01) (Figure 5D) in chondrocytes treated with P2X4-siRNA, no differences were observed in eATP levels in P2X4 silenced cells compared to control cells (Figure 5B). Taken together, these data suggest a redundant system, in which both P2X4 and P2X7 must be inhibited for ATP efflux to be affected.

### Pharmacological inhibitors of ATP efflux do not alter ATP-metabolizing ecto-enzyme activity levels or decrease cell viability

eATP levels can be altered by changes in the activities of the ecto-enzymes that metabolize ATP. Cell damage may also non-specifically increase eATP levels by allowing leakage from injured cells. To verify that these possible effects did not contribute to the action of the pharmacological inhibitors on eATP, we measured activities of ecto-NTPPPH, 5′NT and alkaline phosphatase in the presence and absence of inhibitors, and used the MTT assay as a standard measure of cell injury. None of the inhibitors significantly altered levels of enzyme activities (Table 1). With the exception of flufenamic acid, which was toxic at concentrations greater than 100 μM, no inhibitors or inhibitor combinations significantly decreased cell viability.

## Discussion

These findings support a major and novel role for ANK in eATP efflux in articular chondrocytes. While it is unclear whether ANK itself acts as an ATP channel or regulates such a channel, we propose that the latter possibility is more likely based on our additional findings that suggest roles for P2X7/4 receptors in this process. eATP promotes many of the pathogenic processes resulting in calcium crystal deposition and OA in cartilage. Thus, identifying participants and modulators of ATP efflux may provide insights regarding novel therapies for these diseases.

As is observed in most cell types, chondrocytes release a burst of ATP after exposure to hypotonic media. In chondrocytes, this effect is calcium dependent and is mimicked by a specific chemical agonist of TRPV4, as is true in other cell types [34,40]. While further work will be necessary to conclusively implicate TRPV4 in chondrocyte eATP release, TRPV4 levels are altered in OA chondrocytes, and dysregulation of ATP/PPi efflux could contribute to the excess calcification seen in OA and in TRPV4-deficient mice [22].

The potent effects of ANK silencing in reducing eATP levels confirm and mechanistically extend the important roles of this protein in cartilage homeostasis and disease. ANK levels are increased in OA [41] and CPP crystal-containing cartilage [42], and expression of ANK has been implicated in maintaining the phenotype of healthy chondrocytes [43]. ANK levels are increased with mechanical stimuli in vertebral endplate chondrocytes [44]. We show here that altering levels of ANK is an effective way of manipulating eATP levels in chondrocyte cultures.

Our studies suggest that ANK directly affects eATP efflux. Suppressing ANK protein levels did not result in changes in ATP metabolizing ecto-enzymes. Moreover, the effect of ANK silencing on eATP levels was not mediated by changes in ePPi. As alkaline phosphatase is a marker of the hypertrophic phenotype and levels of alkaline phosphatase activity were unchanged in ANK-silenced cells, we have no evidence to suggest that an altered chondrocyte phenotype is responsible for the changes in eATP levels with ANK manipulation.

The drug, probenecid, acts as a potent inhibitor of both basal and stimulated ATP efflux in chondrocytes. Probenecid may be directly interacting with ANK, as has been hypothesized by Ho *et al*. [9], but may also inhibit hemichannels. We feel that this is an unlikely mechanism for the probenecid effect as no other hemichannel inhibitor reduced eATP efflux. Probenecid also functions as a weak phosphodiesterase inhibitor, but does not appear to act through this mechanism in chondrocytes [35]. The actions of organic anion transporters (OATs) may also be blocked by probenecid. However, the observations that OATs are downregulated by protein kinase C (PKc) [45], and that PKc activation increases chondrocyte eATP levels [8], argue against a likely role for OATs in eATP release. Although plasma levels of probenecid under therapeutic conditions are 10-fold lower than levels typically used in cell culture [46], this drug has a long history of safety and efficacy in patients with gout.

While ANK itself may transport ATP, our findings suggest that P2X7/4 receptors also contribute to eATP release by chondrocytes. Whether these receptors contain a large pore capable of transporting ATP or regulate such a pore is not clear. Our data suggest that, in chondrocytes, a P2X7/4-dependent pore releases PGE_2_ as well as ATP. The lack of effectiveness of the more specific P2X7 inhibitors supports a role for P2X4 in this process, which is further demonstrated by the effect of ivermectin, a relatively specific stimulant of P2X4 receptor-mediated actions. Because reducing levels of P2X4 or P2X7 alone had no effect on eATP efflux, we hypothesize that either P2X4 and/or P2X7 can participate in eATP transport. The redundancy of this system may attest to the importance of eATP efflux in cartilage.

In some cell types, pannexin-1 hemichannels may be activated in response to P2X7 receptor stimulation, and these serve as the conduit for ATP release. However, the ability of P2X7 receptors to facilitate non-selective pore formation is similar in macrophages from wild-type or pannexin-1 knockout mice [47]. In other cell types in which P2X7 receptors participate in eATP release, hemichannel inhibitors behave anomalously [16], and this may be the case in chondrocytes. Our findings differ from those of Garcia and Knight who showed that flufenamic acid reduced eATP release in bovine chondrocytes [12]. Variations in mechanisms among different species, effects of culture conditions and differences in ages of the animals may explain these differences. In a mouse growth-plate chondrocytic cell line, Iwamoto *et al*. showed an important role for pannexin-3 in eATP efflux [48]. Certainly, growth-plate chondrocytes differ from primary articular chondrocytes in many ways. Despite the use of a number of hemichannel inhibitors in a wide range of concentrations, however, we could not demonstrate a clear role for pannexins or connexins in our system.

These studies are not without limitations. Culture models may not fully reproduce the environment that chondrocytes see *in situ*. However, our cells retain all the phenotypic features of highly differentiated chondrocytes [49], and we showed similar behavior in regards to eATP efflux in chondrocytes embedded in an agarose construct. While membrane injury resulting from cell swelling may result in non-specific leakage of cell contents including ATP from the cell, the lack of evidence of toxicity and the specificity of the inhibitor effects makes this highly unlikely. The natural environment of healthy articular chondrocytes is hyperosmolar, and time may be necessary for chondrocytes to adjust to the lower osmolar milieu of culture media [27]. While we allowed cells to acclimatize for 24 hours before these experiments were undertaken, differences in absolute or relative osmolarity may exist between tissue culture models and conditions *in vivo*. We used a brief osmotic stress to elicit eATP efflux and further work will be necessary to explore the long-term effects of various osmotic states on eATP efflux. Last, we were unable to conclusively prove a role for P2X7/4 receptors using silencer technology. Ultimately, studies with mice deficient in one of more of these proteins may be necessary to demonstrate a role for these proteins in chondrocyte ATP efflux. We attempted to minimize concerns about off-target effects of pharmacologic inhibitors by carefully examining toxicity of these agents, as well as testing their actions on other factors impacting eATP levels.

## Conclusion

In summary, we show here that ANK has a central role in eATP release by mature articular chondrocytes, and P2X7/4 receptors may also participate in this process. As eATP has numerous catabolic effects in cartilage and contributes to calcium crystal arthritis, further progress in understanding mechanisms and identifying modulators of ATP release may result in additional therapies for common degenerative diseases of cartilage.

## Abbreviations

5′NT: 5′ nucleotidase; ANK: Progressive ankylosis gene product; BAPTA-AM: Bis-N,N,N',N'-tetraacetic acid-AM; BBG: Brilliant Blue G; BCP: Basic calcium phosphate; CPP: Calcium pyrophosphate; DMEM: Dulbecco’s modified eagle’s medium; eATP: extracellular adenosine triphosphate; ENPP1: Ecto-nucleoside triphosphohydrolase 1; ePPi: Extracellular pyrophosphate; HBSS: Hank’s balanced salt solution; MTT: 3-(4,5-dimethylthiazol-2-yl)-2,5-diphenyltetrazolium bromide; NaOH: Sodium hydroxide: NaPPi: Sodium pyrophosphate; NTPPPH: Nucleoside triphosphate pyrophosphohydrolase; OA: Osteoarthritis; OAT: Organic anion transporter; PGE2: Prostaglandin E_2_; PKc: Protein kinase C; PNPMP: P-nitrophenol thymidine monophosphate; PNPP: P-nitrophenol phosphate; RT-PCR: Reverse transcription polymerase chain reaction; siRNA: Small interfering RNA; TBS: Tris-buffered saline; TRPV4: Transient receptor potential vanilloid 4; VSOAC: Volume-sensitive organic osmolyte and anion channels; VSOR: Volume-sensitive outwardly rectifying channels.

## Competing interests

The authors declare they have no competing interests.

## Authors’ contributions

AKR designed the studies and drafted the manuscript. CMG performed all of the tissue culture experiments, western blotting and the biochemical assays included in this manuscript, assisted with manuscript preparation and drafted the figures. EMF performed all of the RT-PCR procedures including the experiments with siRNA. She also assisted in manuscript preparation, statistical analysis and figure design. MKL carried out the experiments with P2X7 inhibitors and measured PGE_2_ levels. GRD participated in study design and manuscript preparation. LMR assisted with study concept and design and helped to draft the manuscript. All authors read and approved the final manuscript.
